# Pregnancy-associated breast cancer in rural Rwanda: the experience of the Butaro Cancer Center of Excellence

**DOI:** 10.1186/s12885-018-4535-y

**Published:** 2018-06-05

**Authors:** Jean Marie Vianney Dusengimana, Vedaste Hategekimana, Ryan Borg, Bethany Hedt-Gauthier, Neil Gupta, Susan Troyan, Lawrence N. Shulman, Ignace Nzayisenga, Temidayo Fadelu, Tharcisse Mpunga, Lydia E. Pace

**Affiliations:** 1 Partners In Health/Inshuti Mu Buzima, P.O.Box 3432, Kigali, Rwanda; 2 Ministry of Health, Butaro District Hospital, Butaro, Rwanda; 3 Harvard Medical School, Boston, MA USA; 4 Brigham and Women’s Hospital, Boston, MA USA; 5 University of Pennsylvania, Philadelphia, PA USA; 6 Dana Farber Cancer Institute, Boston, MA USA

**Keywords:** Breast cancer, Pregnancy, Breastfeeding, Africa

## Abstract

**Background:**

Breast cancer is the most common malignancy encountered during pregnancy. However, the burden of pregnancy-associated breast cancer (PABC) and subsequent care is understudied in sub-Saharan Africa (SSA). Here, we describe the characteristics, diagnostic delays and treatment of women with PABC seeking care at a rural cancer referral facility in Rwanda.

**Methods:**

Data from female patients aged 18–50 years with pathologically confirmed breast cancer who presented for treatment between July 1, 2012 and February 28, 2014 were retrospectively reviewed. PABC was defined as breast cancer diagnosed in a woman who was pregnant or breastfeeding. Numbers and frequencies are reported for demographic and diagnostic delay variables and Wilcoxon rank sum and Fisher’s exact tests are used to compare characteristics of women with PABC to women with non-PABC at the alpha = 0.05 significance level. Treatment and outcomes are described for women with PABC only.

**Results:**

Of the 117 women with breast cancer, 12 (10.3%) had PABC based on medical record review. The only significant demographic differences were that women with PABC were younger (*p* = 0.006) and more likely to be married (*p* = 0.035) compared to women with non-PABC. There were no significant differences in diagnostic delays or stage at diagnosis between women with PABC and women with non-PABC women. Eleven of the women with PABC received treatment, three had documented treatment delays or modifications due to their pregnancy or breastfeeding, and four stopped breastfeeding to initiate treatment. At the end of the study period, six patients were alive, three were deceased and three patients were lost to follow-up.

**Conclusions:**

PABC was relatively common in our cohort but may have been underreported. Although patients with PABC did not experience greater diagnostic delays, most had treatment modifications, emphasizing the potential value of PABC-specific treatment protocols in SSA. Larger prospective studies of PABC are needed to better understand particular challenges faced by these patients and inform policies and practices to optimize care for women with PABC in Rwanda and similar settings.

**Electronic supplementary material:**

The online version of this article (10.1186/s12885-018-4535-y) contains supplementary material, which is available to authorized users.

## Background

Breast cancer is the most common type of malignancy encountered during pregnancy [1–5]. Despite the growing body of literature on pregnancy-associated breast cancer (PABC), most often defined as breast cancer diagnosed during a pregnancy or within one to two years after delivery with some definitions extending up to five years after delivery [6], the implications of PABC remain poorly understood. Reports on prevalence, optimal management, prognosis and survival of women with PABC versus women with non-PABC are conflicting and this has had repercussions for how PABC is managed [7–12]. While at one time considered to be untreatable, recent reports suggest that mothers and babies can do well following treatment during pregnancy if it is delivered in a timely manner [5, 7]. For operable disease, surgery is considered the best treatment during pregnancy and chemotherapy can be safely delivered in the second and third trimesters [2, 8, 13–16].

Breast cancer is a growing public health concern in low- and middle-income countries (LMICs), including in sub-Saharan Africa (SSA), where both breast cancer incidence and mortality rates are rising [17–20]. Women with breast cancer in SSA are more likely to die of their disease than women with breast cancer in high-income regions in part because of delays in diagnosis leading to advanced stages at presentation [21]. It is likely that the relative burden of PABC is higher in SSA than in North America and Europe because of the region’s higher fertility rates, younger population, and younger median age at breast cancer diagnosis [22–25]. Women with PABC in SSA may face even longer delays to diagnosis and treatment than their counterparts with non-PABC because of low levels of breast cancer awareness, the possibility of breast cancers being misidentified as normal breast modifications that occur during pregnancy or lactation, providers delaying diagnostic workup until after pregnancy or lactation is complete, the competing priorities of pregnant women or new mothers, and the lack of alternatives to breastfeeding which make it difficult for a mother if she is advised to stop for treatment [9, 26–31]. However, despite the likely higher burden of PABC in SSA and the additional challenges women with PABC face, little is known about prevalence, presentation and management of PABC in the region [7, 8, 17, 24, 27].

Rwanda is a small low-income country in East Africa with one of the highest population densities in the continent. Breast cancer is the most common cancer diagnosed in women at national referral hospitals and is the most common adult cancer encountered at the Butaro Cancer Center of Excellence (BCCOE), a specialized cancer facility in the rural northwest [32–36]. Women at BCCOE often present with late stage disease and both patient and system delays are associated with later stage at presentation [21]. In this study, we focus on women with PABC seeking care at BCCOE to better understand their current demographic and clinical characteristics, diagnostic delays, how they are treated and their outcomes at the end of the study period to inform the provision of care for patients with PABC in LMICs.

## Methods

### Study design and setting

This retrospective cohort study included women with breast cancer presenting for treatment at BCCOE. BCCOE was opened by the Rwanda Ministry of Health in July 2012, with the support of the organizations Partners In Health, Dana-Farber Cancer Institute and Brigham and Women’s Hospital in Boston, USA [33]. Patients are referred to BCCOE from across Rwanda and adjacent countries because of the availability and affordability of pathology services, chemotherapy, surgery and clinicians with training in cancer care. As there are no full-time oncologists at BCCOE [37], cancer treatment is facilitated through the implementation of nationally-endorsed protocols based on international standards [33–36]. Medical oncology care is provided by a team of general physicians, internists, pediatricians and nurses who follow the protocols and consult regularly with international oncology specialists. Surgical care is provided by general surgeons. As of July 2017, there was no radiation available in Rwanda.

The existing breast cancer treatment protocols provide simplified algorithms for early stage, locally advanced and metastatic disease. Since there are no specific guidelines for women with PABC, treatment follows general breast cancer protocols but can be modified based on clinician judgment, with expert consultation available from one of the partnering institutions.

### Study population and data collection

The study population included female patients with pathologically confirmed breast cancer who presented at BCCOE between July 1, 2012 and February 28, 2014. Only women between 18 and 50 years old were included in the analysis to allow us to focus on the experiences of women of reproductive age and to increase the comparability of women with PABC to women with non-PABC. Clinical data were extracted from an existing database. This database included information from the medical records, such as cancer diagnosis and staging information, for all BCCOE patients who presented with a breast complaint during the study period. Staging of breast cancer patients at BCCOE was conducted according to the American Joint Committee on Cancer (AJCC) staging system [38], but treatment protocols were based on whether patients fell into simplified categories of “early,” “locally advanced,” or “metastatic” disease. In Table 1 patients are grouped as having non-metastatic and metastatic disease to increase power to explore differences in these two groups. A focused chart review of all patients meeting the study criteria was conducted to confirm who had PABC. For our purposes, PABC was defined as breast cancer diagnosed during pregnancy or when a woman was breastfeeding. Breastfeeding was included within our definition because the date of last delivery was not always documented in cancer patients’ medical records, making it impossible to calculate the precise length of time since delivery. Since the median duration of breastfeeding in Rwanda is approximately 2.5 years [36, 39], we deemed it reasonable to use breastfeeding as a proxy for being within two to three years postpartum [6]. Any patients that met our definition were classified as having PABC, and data on their treatment and outcomes was collected.

**Table 1:** Subset of breast cancer patients with demographic, diagnostic delays and staging data available; comparing women with and without pregnancy associated breast cancer

	PABC^a^ (*N* = 12) n (%) unless otherwise noted	Non-PABC (*N* = 62) n (%) unless otherwise noted	*p*-value
Demographics			
Age - Median (IQR)	37 (35,38)	43 (37, 47)	0.006^c^
Marital Status			
Married/In relationship	11 (91.7)	32 (51.6)	0.035^b^
Widow/Divorced	1 (8.3)	15 (24.2)	
Single	0 (0)	15 (24.2)	
Education level			
None	2 (16.7)	12 (19.4)	0.911^b^
Primary	6 (50.0)	28 (45.2)	
Secondary	3 (25.0)	11 (17.7)	
University	1 (8.3)	11 (17.7)	
Health insurance status			
Mutuelle^d^	9 (75.0)	46 (74.2)	0.879 ^b^
RAMA^e^	3 (25.0)	11 (17.7)	
Other private	0	3 (4.8)	
Not applicable	0	2 (3.2)	
Distance from home to health facility			
Less than 1 h	7 (58.3)	35 (56.5)	> 0.999
1–2 h	3 (25.0)	16 (25.8)	
More than 2 h	2 (16.7)	11 (17.7)	
Care seeking patterns and clinical presentation			
Healthcare provider visited first (*N* = 10 for PABC, *N* = 44 for non-PABC)			
Health center	8 (80.0)	33 (75.0)	0.883^b^
Hospital outside Rwanda	0	2 (4.6)	
Private hospital	2 (20.0)	6 (13.6)	
Referral hospital	0	3 (6.8)	
Time between symptom onset and first presentation to a health facility (days)			
Median (IQR)	109 (6.5, 325.5)	139.5 (28, 402)	0.367^c^
Time between first presentation at health facility and diagnosis (days)			
Median (IQR)	212 (58.5, 362)	145 (59, 328)	0.907^c^
Stage at diagnosis			
Non-metastatic	9 (75.0)	47 (75.8)	> 0.999^b^
Metastatic	3 (25.0)	14 (22.6)	
Unknown	0	1 (1.6)	
Hormone receptor status			
Positive	8 (66.7)	33 (53.2)	0.798 ^b^
Negative	3 (25.0)	23 (37.1)	
Unknown	1 (8.3)	6 (9.7)	
ECOG status			
0	10 (83.3)	41 (66.1)	0.499^b^
1	0	10 (16.1)	
2	1 (8.3)	5 (8.1)	
3	0	0	
Unknown	1 (8.3)	6 (9.7)	

^a^*PABC* Pregnancy associated breast cancer

^b^Fisher’s exact test

^c^Wilcoxon rank sum test

^d^General national medical insurance

^e^*Rwanda Assurance Maladie* – Employed national medical insurance

For the focused chart review, a data collection form designed by the research team included a series of yes/no questions to prompt the data collector to record treatment information and to note any indications of treatment delays or modifications due to PABC (Additional file 1: Appendix 1). The form also asked “Did the patient stop breastfeeding while receiving breast cancer treatment? If yes, why?” Treatment and outcomes data were collected at the time of chart review - March 31, 2015. Outcomes were based on treatment notes documenting patients’ status as of this date and categorized as alive and in care, lost to follow-up and deceased. A patient was considered lost to follow-up if her last recorded clinic visit was six months or more prior to March 31, 2015 and there was no indication in her chart that she had finished treatment.

For detailed information on demographics, diagnostic delays and staging, we consulted a second database developed from surveys conducted as part of a separate study of diagnostic delays [21]. All women ages 21 or older with a breast complaint who were available at the time of survey administration were included in this study. This database was linked to the clinical database using unique patient identification numbers. For the current analysis, we used survey responses from women with pathologically confirmed cancer. Patient delay was calculated as the time between breast symptom onset and first presentation to a doctor or nurse and system delay was calculated as the time between the first visit to a doctor or nurse and the date of the first pathology report confirming breast cancer.

### Analysis

We described demographic characteristics, diagnostic delays and stage at diagnosis for the women ages 21 and older contained in the secondary dataset. We used Wilcoxon rank sum and Fisher’s exact tests to compare characteristics of women with PABC to women with non-PABC at the alpha = 0.05 significance level. We described treatment and outcomes among women with PABC only, indicating any modifications made to treatment due to pregnancy or breastfeeding. Statistical analyses were performed using Stata v.13 (College Station, TX: Stata Corp LP).

## Results

### Characteristics of women with PABC

**Fig. 1:**
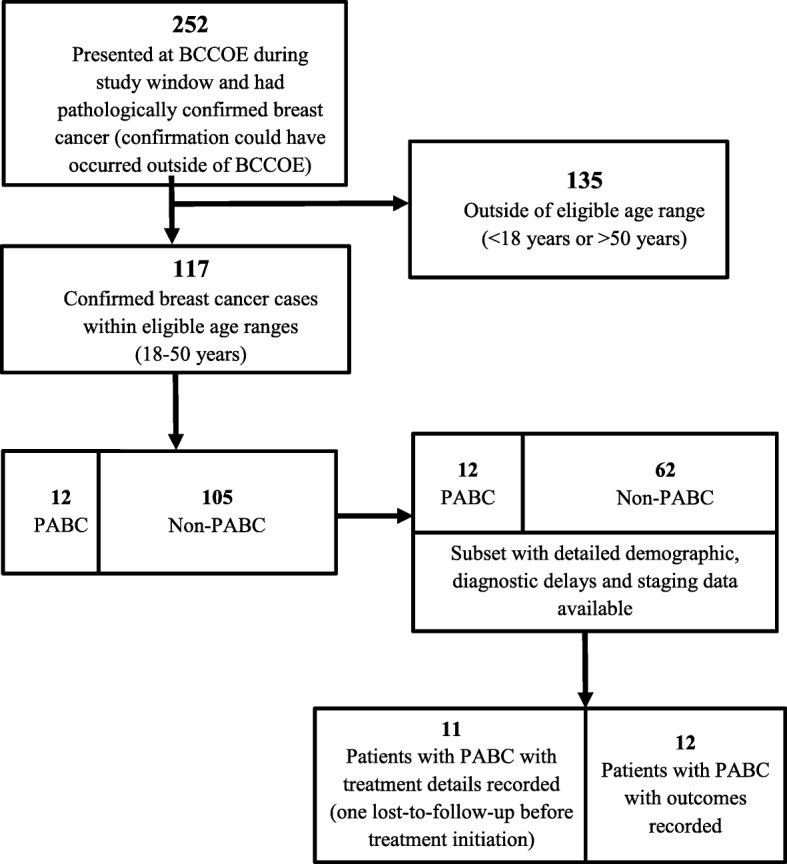
A flowchart describing sample included in PABC analysis

There were 252 women that presented to BCCOE with confirmed breast cancer within the study window, and 117 (46.4%) were between 18 and 50 years old (Fig. 1). Of these 117 patients, 12 (10.3%) met our criteria for having PABC. All 12 (100%) patients with PABC and 62 (59.0%) patients with non-PABC were contained in the secondary dataset (Table 1). The median age for women with PABC was 37 years (interquartile range [IQR]: 35, 38). Most women with PABC were married or in a relationship (*n* = 11, 91.7%) and had a primary school level of education or less (*n* = 6, 50.0%). Nine women (75.0%) had *Mutuelle de Sante*, the national health insurance, and 7 (58.3%) lived within one hour of the nearest health center. The only significant demographic differences between women with PABC and women with non-PABC were that women with PABC were significantly younger (*p* = 0.006) and more likely to be married (*p* = 0.035).

The median time from first onset of symptoms to first visit to a health facility (patient delay) was 109 days (IQR: 6.5, 325.5) for women with PABC and 139.5 days (IQR: 28, 402) for women with non-PABC (*p* = 0.367). The median time from first visit to a health facility and diagnosis (system delay) was 212 days (IQR: 58.5, 362) for women with PABC and 145 days (IQR: 59, 328) for women with non-PABC (*p* = 0.907). Three (25.0%) of the women with PABC had metastatic disease and 8 (66.7%) had tumors that were hormone receptor status positive. We did not identify any significant differences between women with PABC and women with non-PABC in terms of diagnostic delays or stage at presentation (Table 1).

### PABC treatment and outcomes

Among the 12 women identified as having PABC, 3 were pregnant at the time of diagnosis, and 9 were breastfeeding. Eleven patients (91.7%) underwent at least one treatment modality and one woman (who was pregnant at diagnosis) was lost to follow-up before starting treatment (Table 2). As first course of treatment, four (36.4%) women received endocrine therapy, five (45.5%) women received neoadjuvant IV chemotherapy, one (9.1%) woman received endocrine therapy and neoadjuvant IV chemotherapy and one (9.1%) woman underwent a mastectomy. Of the 11 patients who received any treatment, 3 women (27.2%) had indication of a delay or modification in treatment due to pregnancy or breastfeeding. Of these, two women delayed the start of treatment because of pregnancy and one woman delayed the start of treatment because she was breastfeeding. Four women (36.3%) were documented to have stopped breastfeeding in order to start treatment. One other woman stopped breastfeeding during her treatment, but the reasons were not specified. For the other 3 women who were breastfeeding at diagnosis and received treatment, cessation or continuation of breastfeeding was not documented in the record.

**Table 2:** Treatment of women with PABC (*N* = 12)

	n	%
Type of PABC (*n* = 12)		
Pregnant at diagnosis	3	25.0
Breastfeeding at diagnosis	9	75.0
Patient received any treatment		
Yes	11	91.7
No (lost to follow-up prior to treatment)	1	8.3
Limited to individuals who received treatment (*n* = 11)		
Type of treatment initially received		
Endocrine therapy	4	36.4
Neoadjuvant IV chemotherapy	5	45.5
Endocrine therapy and neoadjuvant IV chemotherapy	1	9.1
Mastectomy	1	9.1
Any indication of treatment delays or modifications due to pregnancy or breastfeeding?		
No	9	81.8
Yes	3	27.2
Delayed start of treatment because of pregnancy	2	18.2
Delayed start of treatment because of breastfeeding	1	9.1
Stopped breastfeeding to start treatment	4	36.4

As of March 31, 2015, six (50.0%) of the women with PABC were alive, of which four (66.7%) were still receiving chemotherapy or endocrine therapy, one (16.7%) was on palliative care and one (16.7%) completed treatment. Three (25.0%) patients were deceased, all of which had metastatic disease at diagnosis and three (25.0%) patients were lost to follow-up.

## Discussion

We identified 12 women with PABC who enrolled at BCCOE between July 1, 2012 and February 28, 2014, representing 10.3% of women ages 18–50 years diagnosed with breast cancer. The proportion of women with PABC is somewhat higher than rates documented in Europe (for example, 7.1% of women under 45 with breast cancer in Sweden [40]) but lower than rates documented in other sub-Saharan sites (for example, 21.2% of premenopausal women with breast cancer at a Nigerian hospital were diagnosed during or within two years after pregnancy [6]). However, we believe that our study underestimates the true rate of PABC prevalence at BCCOE since pregnancy and breastfeeding status in our cancer patients is not routinely documented. Women with PABC did experience considerable delays prior to diagnosis, with a median time of 109 days from symptom onset to first visit at a health facility, and 212 days from first visit to a health facility to initial diagnosis.

Based on our review of the literature, we hypothesized that women with PABC would experience longer diagnostic delays and as a result have more advanced disease at diagnosis [5, 25, 30, 41]. We did not find this in our population, which may be attributable to our small sample size which limited our power to detect differences between women with and without PABC. However, since long diagnostic delays have been observed in patients with PABC in other settings [3, 9, 27, 28, 42–44] and because most patients with breast cancer at BCCOE experience long diagnostic delays regardless of pregnancy and lactation status, [21] educational interventions for patients and providers in Rwanda are important for promoting earlier breast cancer detection, and should include the fact that breast cancer can occur during pregnancy and lactation. Leveraging existing health care interactions that occur for pregnant and breastfeeding women could make pregnancy an opportunity for early diagnosis instead of a risk factor for delay and late stage presentation [20, 21, 45, 46].

In our study, three women with PABC had documented modifications made to their treatment plan because of pregnancy or breastfeeding. Treatment considerations in PABC are complex and often require an individualized approach, though key principles (such as avoiding chemotherapy in the first trimester) should be disseminated in breast cancer treatment protocols [31]. Effective and safe treatment for patients with PABC also requires multidisciplinary collaboration [31]. At BCCOE, this often entailed email or phone consultations with surgical or medical oncologists and obstetricians based in Rwanda’s capital city or in the United States. Further investigations into how prognosis is associated with delayed or modified treatment because of pregnancy or breastfeeding in our setting are needed.

In addition to the psychosocial challenges faced by any young woman with a cancer diagnosis, patients in SSA and other low-income regions face particular challenges when diagnosed with PABC. Four patients stopped breastfeeding because of cancer treatment. While this can be important for the health of the baby, stopping breastfeeding is a particular challenge in Rwanda because of the expense of appropriate alternatives (e.g. infant formula) with resulting consequences for babies’ health. These challenges must be addressed by oncology programs in Rwanda and similar settings, through financial support for procurement of formula. Additionally, for women who are pregnant at diagnosis, therapeutic abortion may be the preferred choice, and in some settings therapeutic abortion may be legally, logistically, and culturally difficult to pursue. Research focused specifically on managing PABC in SSA and similar low-resource settings that have limited diagnostic and treatment capacity will be vital to understand the complex needs of these patients and address the increasing burden of the disease [40].

This study has several limitations. First, the small number of women with PABC limited our ability to detect differences in delays and stage at presentation. Second, PABC status is likely underreported because pregnancy or breastfeeding status is not systematically documented in our medical record system. Our findings underscore the importance of comprehensive and systematic data collection and management systems in LMICs in order to accurately assess patient diagnoses, treatment and outcomes. Third, due to a lack of sufficient follow-up time to have key outcomes data, we did not compare outcomes of patients with PABC patients to outcomes of patients with non-PABC. We plan to study this in the future, when we have more individuals and longer follow-up. Our findings are not necessarily generalizable to other settings in sub-Saharan Africa, particularly facilities that are not rural, or hospitals that are not cancer referral facilities. We suspect that in settings with more barriers to cancer care, the challenges facing women with PABC could be even more acute. Further research both in our settings and in others will help identify the range of issues that need to be addressed across the region.

## Conclusion

This study highlights that PABC is an important clinical challenge among patients diagnosed with breast cancer in Rwanda. As the burden of breast cancer rises in LMICs, further research is needed to illuminate the scope of this issue, understand its medical, cultural and psychosocial implications for patients, and help develop context-specific management protocols in sub-Saharan Africa.

## Additional file

Additional files 1: Appendix 1, Portion of data collection form designed by researchers to record delays, deviations and modifications in treatment for PABC cohort**.** This form was used during the medical record abstraction process to identify treatment delays and modifications due to pregnancy or breastfeeding for patients with PABC. (DOCX 17 kb)

## Abbreviations

AJCC: American Joint Committee on Cancer
BCCOE: Butaro Cancer Center of Excellence
IQR: Interquartile range
LMICs: Low- and middle-income countries
PABC: Pregnancy-associated breast cancer
RAMA: *Rwanda Assurance Maladie* (Employed National Medical Insurance)
SSA: Sub-Saharan Africa
USA: United States of America
