# Supplementary material for: Pregnancy-associated breast cancer in rural Rwanda: the experience of the Butaro Cancer Center of Excellence
Source: BMC Cancer. 2018 Jun 5;18:634. doi: 10.1186/s12885-018-4535-y (PMC5987575; doi:10.1186/s12885-018-4535-y)
Supplement: Supplementary file 1 — Appendix 1, Portion of data collection form designed by researchers to record delays, deviations and modifications in treatment for PABC cohort. This form was used during the medical record abstraction process to identify treatment delays and modifications due to pregnancy or breastfeeding for patients with PABC. (DOCX 17 kb) [file 12885_2018_4535_MOESM1_ESM.docx]

| **Type of Treatment Initially Given (mark all that are documented)** | | | | | |
| --- | --- | --- | --- | --- | --- |
| 8. Treatment Type | *Endocrine therapy/ hormonotherapy* | *IV chemotherapy*  *Neoadjuvant* | *IV chemotherapy*  *Adjuvant* | *Mastectomy* | *Radiotherapy* |
| *8a. Performed?* | 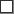 Yes  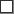 No | 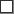 Yes  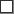 No | 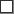 Yes  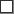 No | 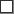 Yes  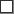 No | 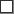 Yes  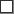 No |
| *8b. Start date* |  |  |  |  |  |
| *8c. End date* |  |  |  |  |  |
| *8d. If started, did delays occur?* | 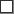 Yes  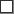 No  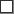 Not documented | 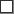 Yes  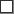 No  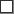 Not documented | 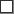 Yes  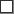 No  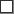 Not documented | 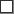 Yes  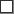 No  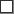 Not documented | 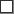 Yes  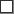 No  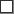 Not documented |
| *8e.Delay reasons* | 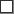 Pregnancy  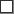 Breastfeeding  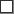 Other | 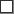 Pregnancy  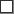 Breastfeeding  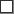 Other | 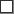 Pregnancy  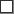 Breastfeeding  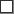 Other | 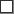 Pregnancy  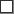 Breastfeeding  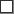 Other | 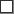 Pregnancy  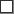 Breastfeeding  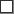 Other |
| *8f. If not started, was it because of PABC?* | 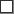 Yes  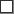 No  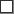 Not documented | 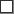 Yes  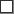 No  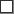 Not documented | 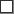 Yes  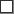 No  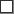 Not documented | 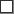 Yes  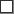 No  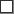 Not documented | 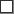 Yes  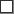 No  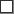 Not documented |
| *8g. Other reasons for not starting* |  |  |  |  |  |
| **Treatment Deviation** | | | | | |
| 9. Is there evidence that the treatment was modified from the recommended protocol? 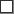 Yes _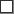_ No  9a. If yes, was the treatment modified because of pregnancy? 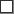 Yes _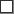_ No  9b. If yes, was the treatment modified because of breastfeeding? 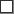 Yes _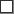_ No | | | | | |

**Additional file 1 Appendix 1: Portion of data collection form designed by researchers to record delays, deviations and modifications in treatment for PABC cohort**
